# Combined pattern of childhood psycho-behavioral characteristics in patients with schizophrenia: a retrospective study in Japan

**DOI:** 10.1186/s12888-021-03049-w

**Published:** 2021-01-26

**Authors:** Yukiko Hamasaki, Takao Nakayama, Takatoshi Hikida, Toshiya Murai

**Affiliations:** 1 Faculty of Contemporary Society, Kyoto Women’s University, 35, Kitahiyoshi-cho, Imakumano, Higashiyama-ku, Kyoto, 605-8501 Japan; 2 Shigasato Hospital, 1-18-41 Shigasato, Otsu, Shiga 520-0006 Japan; 3 Laboratory for Advanced Brain Functions, Institute for Protein Research, Osaka University, 3-2 Yamadaoka, Suita, Osaka, 565-0871 Japan; 4 Department of Psychiatry, Kyoto University Graduate School of Medicine, Yoshida-Konoe-cho, Sakyo-ku, Kyoto, 606-8501 Japan

**Keywords:** Schizophrenia, Prodrome, Psychosis, Child behavior checklist, Childhood characteristics, Early identification

## Abstract

**Background:**

Although epidemiological and genetic studies have provided scientific evidence that places schizophrenia into the framework of early neurodevelopmental disorders, the psycho-behavioral characteristics of children that later go on to develop schizophrenia have not been sufficiently clarified. This study aimed to retrospectively identify characteristics specific to patients with schizophrenia during childhood via their guardians’ reporting of these characteristics.

**Methods:**

Participants included 54 outpatients with schizophrenia in their twenties who fulfilled DSM-IV-TR criteria. Additionally, 192 normal healthy subjects participated as sex- and age-matched controls. The guardians of all participants were recruited to rate participants’ childhood characteristics from 6 to 8 years of age on a modified version of the Child Behavior Checklist (CBCL), which was used as a retrospective assessment questionnaire. Using *t*-tests, logistic regression, and Receiver Operating Characteristic (ROC) curve analysis, we estimated the psycho-behavioral characteristics specific to schizophrenia during childhood. Using the obtained logistic regression model, we prototyped a risk-predicting algorithm based on the CBCL scores.

**Results:**

Among the eight CBCL subscale *t*-scores, “withdrawn” (*p* = 0.002), “thought problems” (*p* = 0.001), and “lack of aggressive behavior” (*p* = 0.002) were each significantly associated with the later diagnosis of schizophrenia, although none of these mean scores were in the clinical range at the time of childhood. The algorithm of the logistic regression model, based on eight CBCL subscales, had an area under the ROC curve of 82.8% (95% CI: 76–89%), which indicated that this algorithm’s prediction of late development of schizophrenia has moderate accuracy.

**Conclusions:**

The results suggest that according to guardian reports, participants showed psycho-behavioral characteristics during childhood, different to those of healthy controls, which could be predictive of the later development of schizophrenia. Our newly developed algorithm is available to use in future studies to further test its validity.

## Background

Schizophrenia is a psychiatric disorder that affects a relatively small portion of the population, approximately 20 million people worldwide [1], but its manifestation can be incredibly debilitating. If we were better able to predict who might manifest as schizophrenic in adolescence or adulthood, then intervention strategies could be applied to try to prevent or delay the onset of the disorder. The long-held premise that schizophrenia occurs after a period of normal mental development [2] has been challenged recently. For instance, adolescent psychotic patients were often treated in pediatric services for various psychosomatic symptoms before receiving a psychiatric consultation as adolescents [3]. As the direct relationship between “duration of untreated psychosis” and “poor prognosis” has been elucidated, early intervention is crucial [4, 5].

In order to identify children who will eventually develop schizophrenia we will rely on observations of the prodromal phase of the illness. Nearly 90 years after Mayer-Gross first proposed the prodrome concept [6], attention to this concept is increasing once more. The prodrome may be defined as the group of symptoms that indicates the continuous transition to a disorder [7, 8]. Huber identified poor social functioning and cognitive problems as the basic symptoms in the prodrome of schizophrenia [9]. The prodrome period can also be described as the time between the first onset of unusual behavior or noticeable symptoms to the first signs of psychosis [10].

Despite few reports on children’s prodromal symptoms, it has been pointed out that the age of onset, when prodromal symptoms first appear for schizophrenia, is at least 11 years [11]. It is logical to presume that schizophrenia may start to develop, in the form of prodromal signs, even earlier in childhood as it is considered a neurodevelopmental disorder. Research has linked its occurrence to pregnancy and birth complications, perinatal viral exposure, and winter birth, to name a few [12–16].

Studies show extensive brain volume changes from the first psychotic episode of schizophrenia [17]; such changes may even occur during the transition to psychosis [18–22]. The occurrence of major anatomical changes early during the clinically identifiable course of the disease highlights the need for the identification of early prodromal signs. Psychotic-like experiences, which are reported by 15% of adolescents [23], predicted the onset of subsequent psychosis at a high rate [11]. Around 35% of patients who met the Structured Interview for Prodrome Syndromes criteria (e.g., unusual thought content, suspicion/paranoia, perceptual anomalies, grandiosity, and disorganized communication) experienced full psychosis onset within two and a half years [24]. A systematic review of studies attempting to create prognostic models identified a conversion to psychosis rate of 27% on average [25], while another systematic review focusing on ages below 18 reported the 2-year conversion to psychosis rate at a maximum of 21% [26].

Schizophrenia is a psychiatric disorder in particular need of early intervention strategies, which necessitates a valid way to assess who is at risk of developing it. Clinical tools for identifying psychosis risk groups have been developed, mainly in English- and German-speaking countries [27–31]. For example, the Prodromal Questionnaire [30], the Bonn Scale for the Assessment of Basic Symptoms [27], and the Comprehensive Assessment of At-Risk Mental States have all been developed [32]. However, objective biomarkers for psychotic risk group screening have not yet been identified, and current clinical risk identification often relies on the subjective judgement of symptoms by clinicians. For this reason, the accuracy of risk identification inevitably varies. Additionally, prodromal state research and intervention trials have often not included children; meaning that methods for identifying at-risk children have not yet been developed, as previous studies have assumed that prodromal psychopathology does not exist in childhood.

Tor et al. studied the neuropsychological profile of at-risk for psychosis children and adolescents (aged 10–17) [33]. They found this group showed lower general performance in intelligence, executive functioning, and attention compared to healthy controls. A systematic review in 2017 also found that clinically high-risk children and adolescents show lower general intelligence [26]. Retrospective epidemiological studies as far back as the 1970’s have looked for predictive characteristics in childhood, but could not find much with the sensitivity of the tests of the day [34–37]. As a result, studies of childhood prodromal signs of schizophrenia have been largely neglected in the modern literature.

Prospective studies, such as long-term longitudinal research on genetically high-risk children whose mothers had schizophrenia [38] and birth cohort studies [39–41] have also been conducted. These studies indicated that common characteristics of at-risk groups in early childhood (6–8 years) include isolated tendencies, poor social functioning, delayed motor and language development, among others, none of which are unique to schizophrenia. One prospective study conducted by Bolhuis et al. analyzed child behavior at 3 and 6 years old and found that behavioral problems associated with anxiety, depression, and aggression were predictive of psychotic-like experiences at age 10 [42].

The age group 6–8 years is an often neglected time of childhood to be studied. Kolvin et al. reported a biphasic distribution of the age of onset of mental disorders, with developmental disabilities becoming apparent before age 5 and schizophrenia-related disabilities occurring after age 9 [43, 44]. Cognitive abnormalities generally only appear after age 10 [37]. The 6–8 year old group is therefore important to investigate prodromal symptoms of schizophrenia.

To date, none of the early signs reported in studies are specific to schizophrenia, but identifying a specific pattern of characteristics could be useful to predict later development of schizophrenia and identify at-risk children. Therefore, we designed a retrospective clinical epidemiological study of patients with schizophrenia using the Child Behavior Checklist (CBCL) to assess possible behavioral alterations in children (6–8 years old) that could be used to develop a pediatric screening system.

We consider the CBCL to be effective for extracting early characteristics that may be prodromal of schizophrenia as it has been used extensively in the literature to predict psychiatric disorders meeting DSM criteria [42, 45, 46].

A systematic review of the models that aim at predicting the transition to psychosis were broadly found to have poor methodology and reporting of results [25]. Thus, there is a need for better prediction tools, especially those that can be utilized with children. This study aimed to develop a risk-predicting algorithm for identifying children that would benefit from early intervention strategies to reduce the risk of psychosis. The present study is also novel in examining the possibility of extending the prodromal concept to childhood.

## Methods

### Study design and participants

A total of 54 outpatients in their twenties who fulfilled DSM-IV-TR criteria for schizophrenia (30 males; 24 females; age range 20–29 years, DSM codes, 295.10, 295.20, 295.30, 295.60, or 295.90) were recruited (Table 1). Schizophrenia was diagnosed at the clinic by the treating physician and additionally as part of the research protocol. All patients were screened with the Structured Clinical Interview for DSM-IV-TR Disorders [47]; exclusion criteria included current or past mental disorders due to general physical disease on axis III or dependence on alcohol or any illicit substances (DSM codes 303.90, 304.40, 304.30, 304.20, 304.50, 304.00). Acute state schizophrenia patients were also excluded as obtaining consent from these patients is challenging. A total of 192 healthy volunteers (98 males; 94 females; age range: 20–29 years) with no current or past psychiatric history, or any of the exclusion criteria listed above, were used as sex- and age-matched controls (Table 1). Once the patients and healthy volunteers were assessed, their guardians were recruited to provide retrospective information for the study. One guardian or both together completed the CBCL, either at home or at the hospital, creating one record per child. Guardians unable to complete the questionnaire due to intellectual disability or psychotic state were excluded (one case only).

**Table 1:** Demographic Characteristics of Patients with Schizophrenia and Controls^a^

	Schizophrenia	Controls
Sex (M/F)^b^	54 (30/24) 55.5% males	192 (98/94) 51.0% males
Age (in years)^b^	24.1 ± 3.8	24.0 ± 2.8
Duration of illness (in years)^c^	3.78 ± 2.89	–
Age of onset (in years)	20.25 ± 3.63 (min 14, max 27)	–

^a^Mean ± SDs shown unless otherwise stated

^b^No significant difference between the two groups

^c^Years from schizophrenia diagnosis to participation

The outpatients were recruited from a psychiatric university hospital and psychiatric district hospital in the Kansai area between December, 2014 and March, 2017. If the inclusion criteria were satisfied, eligible participants and their parents provided written informed consent after receiving a full explanation of the study. This study was approved by the Ethics committee of Kyoto Women’s University in November 2014 (approval number: 26–21, 2014) and was carried out in accordance with the latest version of the Declaration of Helsinki.

### Assessments

To evaluate the possible prodromal state, we administered the CBCL/4–18 [48], which assesses children’s characteristics within the 6- to 8-year-old period. Using a modified version of the CBCL – as a retrospective assessment questionnaire – we asked the guardians of the patients with schizophrenia and the guardians of the control subjects to rate their children’s behaviors at 6–8 years old. All items were completed by the guardian(s).

The CBCL is a checklist developed by Achenbach et al. that comprehensively evaluates a child’s emotional and behavioral problems [48]. The CBCL/4–18 consists of social competence and problem scales. In the present study, we only used the problem scales as these could be easily digitized for statistical processing. Using the raw scores of the 118 problem items, the scores of 11 subscales were calculated: eight syndrome subscales (withdrawn, somatic complaints, anxious/depressed, social problems, problematic thoughts, attention problems, delinquent behavior, and aggressive behavior) and three summary scales (internalizing scale, externalizing scale, and total score). The CBCL’s scales have consistency, transcending national and cultural differences, and their reliability and validity have been verified in numerous countries [49, 50]. Standard values vary according to country [49–51], and the scores of the 11 scales are converted into *t*-scores based on these standard values. Standardized *t*-scores yield easy international comparisons, making the CBCL a major research tool that is widely used in retrospective [51–53], cohort [54], and meta-analysis [55] studies that examine psychiatric symptoms in childhood and adolescence. In the present study, we used the total score and eight syndrome subscales to elucidate psycho-behavioral characteristics that predict later development of schizophrenia.

A new version of the CBCL/4–18 is currently being revised, the CBCL/6–18 [56]. However, as the current study is a retrospective survey designed to evaluate the condition of participants from roughly 10 to 20 years ago, we decided to use the CBCL/4–18, which can calculate *t*-scores using the standardized values available at that time.

### Statistical analysis

First, *t*-tests for the CBCL total score and eight subscale scores were conducted to investigate differences in psycho-behavioral characteristics between the schizophrenia and control groups. A post-hoc power calculation was conducted to see whether our sample size was adequate for detecting weak effects. The effect size (Cohen’s d) and power (1-β) were calculated. We considered d > 0.2 as small, 0.5 as medium, and 0.8 as large effect size. Power (1-β) > 0.8 was deemed sufficient. Since one group had a larger population than the other, we performed Levene’s test for homoscedasticity to test the variances of the two populations. Our independent variables were normally distributed within each group and passed the homogeneity of variance test. Second, we performed a logistic regression analysis with the eight CBCL subscale *t*-scores to elucidate the predictors of schizophrenia. Third, in order to evaluate goodness-of-fit for the logistic regression model, the Cox-Snell & Nagelkerke R-square value and discrimination accuracy were obtained. The goodness-of-fit test of Hosmer and Lemeshow was also performed. Lastly, receiver operating characteristics (ROC) curve analysis was used to assess the sensitivity and specificity of our logistic regression model in predicting the onset of schizophrenia. The area under the ROC curve (AUC) was computed using non-parametric trapezoids. All analyses were performed using SPSS Version 22 for Windows. The level of significance was set at *p* < 0.05.

## Results

### Psycho-behavioral characteristics during the age period of 6–8 years

The mean CBCL total score in patients with schizophrenia was significantly higher compared to that of control subjects (*p* < 0.01, *d* > 0.2, 1-β > 0.8), though both means were in the clinically normal range (Table 2). Comparisons using *t*-tests also indicated that patients showed significantly higher mean scores (*p* < 0.01, *d* > 0.5, 1-β > 0.8) than control subjects on “Withdrawn,” “Anxious/Depressed,” “Social Problems,” “Thought problems,” and “Attention Problems,” and a significantly lower mean score on “Aggressive Behavior” (*p* < 0.05, *d* > 0.2, 1-β > 0.8; Table 2). These characteristics were considered normal, as all mean scores were under the clinical and borderline score ranges but could still be of a predictive nature.

**Table 2:** Comparison of Mean CBCL Total Score and Subscale *t*-scores between the Schizophrenia and Control Groups^a^

	Schizophrenia	Control	t-value	*p*	*d*^b^	(1-β)^c^
	Mean ± SD (SE)	Mean ± SD (SE)				
Summary scale						
Total score^d^	51.24 ± 11.22(1.52)	46.21 ± 10.54(0.76)	3.04	0.003**	0.47	0.84*
Eight syndrome subscales^e^						
Withdrawn	59.25 ± 9.23(1.25)	53.66 ± 6.08(0.43)	4.20	< 0.001***	0.81	0.97*
Somatic complaints	53.50 ± 6.61(0.90)	52.88 ± 5.72(0.41)	0.67	0.502	0.10	0.09
Anxious/Depressed	56.27 ± 6.91(0.94)	52.29 ± 4.65(0.33)	3.98	< 0.001***	0.76	0.96*
Social problems	56.27 ± 8.11(1.10)	51.93 ± 4.50(0.32)	3.77	< 0.001***	0.79	0.93*
Thought problems	54.00 ± 7.37(1.00)	50.36 ± 1.40(0.10)	3.60	0.001**	1.00	0.89*
Attention problems	55.90 ± 7.95(1.08)	51.98 ± 4.33(0.31)	3.48	0.001**	0.74	0.89*
Delinquent behavior	52.98 ± 5.70(0.77)	53.26 ± 7.62(0.55)	−0.25	0.799	0.04	0.06
Aggressive behavior	51.09 ± 2.96(0.40)	52.78 ± 7.57(0.54)	−1.60	0.014*	0.25	0.95*

^a^*t*-test comparisons, *p < 0.05, **p < 0.01, ***p < 0.001

^b^Effect size (Cohen’s *d*)

^c^Power analysis, *Power(1-β) > 0.8

^d^Clinical range ≧64.64 > borderline range of total score > 59. Normal range≦59.

^e^Clinical range ≧71. 71 > borderline range of syndrome subscales > 66. Normal range≦66

### Predictors of schizophrenia for 6- to 8-year-olds

The logistic regression analysis using the eight CBCL subscale *t*-scores revealed that the withdrawal, thinking problems, and aggressive behavior subscales each significantly contributed to the discrimination of both groups (Table 3). That is, at ages 6–8, the presence of withdrawal, thinking problems, and lack of aggressive behavior could be predictive of schizophrenia onset.

**Table 3:** Logistic Regression Analysis Predicting the Likelihood of Schizophrenia^a^

CBCL syndrome subscale	B	SE	***p***	OR	95% CI for OR	
					Lower	Upper
Withdrawn	.119	0.038	0.002**	1.127	1.045	1.215
Somatic complaints	−.066	0.040	0.095	.936	.866	1.012
Anxious/Depressed	.031	0.044	0.479	1.032	.946	1.125
Social problems	.035	0.055	0.521	1.036	.931	1.152
Thought problems	.279	0.084	0.001**	1.322	1.122	1.558
Attention problem	.129	0.069	0.061	1.137	.994	1.301
Delinquent behavior	−.011	0.065	0.865	.989	.871	1.123
Aggressive behavior	−.428	0.141	0.002**	.652	.494	.860

** *p* < 0.01

^a^Logistic regression model statistics: Cox-Snell *R*^*2*^ = 0.286, Nagelkerke *R*^*2*^ = 0.439. The goodness-of-fit test of Hosmer and Lemeshow: χ^2^ = 2.820, df = 7, *p* = 0.901. Discrimination accuracy = 85.5%. CBCL syndrome scales were all entered into the model as independent variables

### Receiver operating characteristics (ROC) curve analysis

The ROC curve showed that the algorithm of this logistic regression model had an AUC of 82.8% (95%, CI: 76–89%) (Fig. 2), indicating moderate accuracy.

## Discussion

### Psycho-behavioral characteristics in 6–8-year-old children who later develop schizophrenia

This is the first study, to our knowledge, that identifies specific characteristics of 6–8-year-old children who later in life developed schizophrenia using the CBCL subscales. Therefore, it is an important step towards extending the concept of prodromal signs to this younger age group, which historically has been less studied due to the many difficulties associated with studying children.

**Fig. 1:**
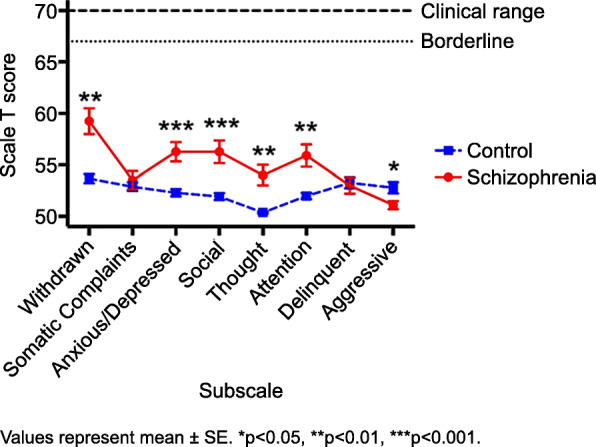
Comparison of CBCL Subscale t-scores between the Schizophrenia and Control Groups

Our comparisons of mean CBCL total scores and subscale *t*-scores between the schizophrenia and control groups (Table 2, Fig. 1) showed that the schizophrenic group was significantly different from the control group in measures of withdrawal, anxiety/depression, social problems, thought problems, attention problems, and aggressive behavior. Withdrawal, impairment in role functioning, and poverty of content of speech have all been listed as criteria for schizophrenia prodrome [10]. Decreased aggressive behavior has been found in other studies in the form of passive involvement in bullying and acceptance of violence from adults [23]. However, Hastings et al. found that individuals who were highly aggressive but also highly withdrawn were at greater risk for other psychosis-spectrum diagnoses [57].

Importantly, all significantly different measures in the schizophrenia group were still within the clinically normal range and, as such, cannot be viewed as symptoms in any way; however, they suggest a potential predictive trend or could even be considered prodromal signs. The goal of identifying prodromal signs is to be able to recognize them as soon as they start to deviate from typical development. Research by Hameed et al. showed that a decline in social and communication skills, rather than persistent impairment in these skills, in children measured annually from 6 months to three and a half years is associated with psychotic experiences at age 12 [58]. If static measures are not as predictive of risk of psychosis as a decline in abilities, then perhaps the reduced abilities seen in this schizophrenic cohort compared to the controls could be indicative of a decline that is still within the clinically normal range, but may worsen with age until it becomes diagnostically significant. A longitudinal study of children who are genetically predisposed to schizophrenia would be required to elucidate how these measures develop as they grow up.

**Fig. 2:**
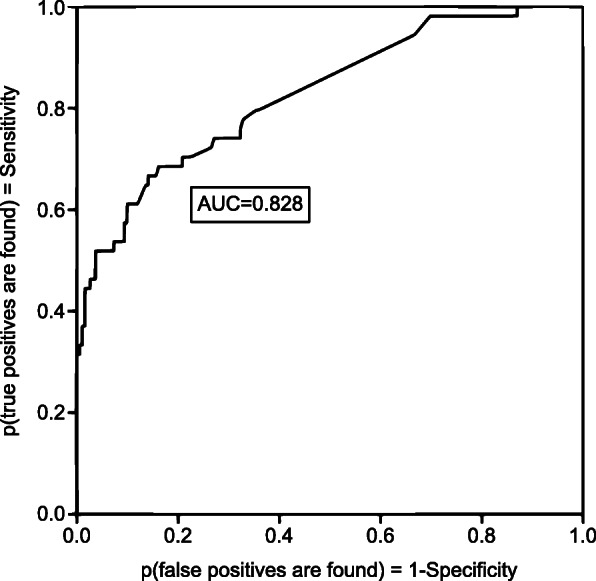
ROC Curve for Binary Classification of the Logistic Regression Model

Our logistic regression model (Table 3, Fig. 2) had sufficient discriminatory power at AUC 0.828 and identified three of the eight subscales as being possibly predictive of the likelihood of schizophrenia. These included withdrawal, thought problems, and a lack of aggressive behavior. The combination of these three psycho-behavioral signs could be utilized to effectively identify children at risk of developing psychosis.

However, Simeonova et al. reported that the CBCL does not seem to be a good predictor for adolescents who have developed a psychotic disorder versus high-risk adolescents who did not develop a psychotic disorder [59]. This discrepancy could be due to age differences between the subjects of both studies. Indeed, retrospective and cohort studies of schizophrenia have reported that CBCL characteristics vary significantly between childhood and adolescence [51–54]. In a meta-analysis, Matheson et al. reported that childhood social withdrawal, as seen in the current study, was significantly higher in adults with schizophrenia than in control adults, which may be a prodromal sign of schizophrenia [55]. Conversely, Miller et al. found that the parents of a high-risk group who developed psychosis reported higher scores in aggressive and delinquent behaviors [52], whereas in our sample aggressive behaviors were decreased in the schizophrenic group. However, such characteristics occurring between the ages of 13 and 16 predicted the onset of psychosis, but not before the age of 13. To further clarify this, childhood and adolescent markers should be examined in a longitudinal study, specifically in terms of brain maturation during periods of increased risk of psychosis [60].

A serious limitation of the current study is that only 54 patients with schizophrenia were recruited. To secure the scientific validity of these results, it is necessary to increase the sample size through ongoing studies. Furthermore, this was a retrospective study in which subjects’ guardians answered questions about the subject’s childhood. Therefore, recall bias likely occurred, with significant ramifications for reliability. There is also the confounding effect of childhood adversities, such as divorce, physical or sexual maltreatment, or moving schools. Bolhuis et al. found that childhood adversities were associated with psychotic-like experiences, which remained significant even after adjustment for comorbid psychiatric problems [42]. Psychological diagnoses were not obtained for the parents of the participants and neither was data on family structure, which may have been indicative of childhood adversity.

### Using CBCL subscales scores to create a risk-prediction algorithm

Based on the obtained logistic regression model, we prototyped a risk-predicting algorithm called the Child Psychosis-risk Screening System (CPSS), available in an interactive web system (https://nakayama-lab.japanwest.cloudapp.azure.com/prototype/), with the aim of facilitating the assessment of children’s risk of developing schizophrenia. The CPSS algorithm requires the use of children’s CBCL scores. The CBCL is a widely accessible, simple questionnaire tool that can be used during preliminary examination in pediatric clinics [56]. Researchers could then simply input the CBCL subscale *t*-scores into the CPSS algorithm to quickly determine the predictive likelihood of schizophrenia for the specific child. Importantly, this algorithm is very much in development and cannot yet claim to have high predictive value, as it still requires extensive verification. That is, the CPSS algorithm is preliminary and not yet conclusive.

We cannot rule out that the identified psycho-behavioral characteristics may have been influenced by sociocultural factors, and international comparative studies will be needed to confirm whether these are universal characteristics. Furthermore, we have not investigated whether they differ from childhood characteristics present in other mental disorders. Finally, we did not examine differences in other characteristics (e.g., IQ) between high-risk individuals who developed schizophrenia and those who did not. In the future, research with larger cohorts is needed to test the validity of the CPSS algorithm and its utility as a screening tool. Longitudinal studies have suggested the need for multiple-domain models of schizophrenia. Research shows that risk for schizophrenia as well as other psychosis-spectrum diagnoses in adulthood is multi-determined, highlighting the need for studying the interactive childhood factors that precede and predict future disorders [57].

The clinical application of screening tools will need to be carefully considered in further research, especially in conjunction with their risks and benefits for early intervention strategies. It is important to carefully consider the usefulness of categorizing high-risk groups (e.g., attenuation of psychosocial stressors experienced by the child, early intervention) and the adverse effects of categorization (e.g., diagnostic trauma, discrimination, risk of false positives, abandonment of positive education, and support). The cost-effectiveness of screening and intervention plans based on predictive childhood characteristics is significant [61]. Also, identifying at-risk groups early in childhood would benefit longer-term studies.

Furthermore, retrospective childhood assessments using CPSS may be useful for predicting the onset of psychosis in adolescent at-risk patients and for assisting with difficulties diagnosing first-onset patients. Longitudinal observations of high-risk children in clinical settings could help elucidate the dynamic system of the critical period from a pre-psychotic state to symptom onset. For example, it may be possible to separate what is a basic vulnerability from what becomes a trigger for the onset of psychosis (e.g., maternal deprivation or bullying) and to observe behavioral, neurophysiological, and functional brain changes over time starting from childhood. Longitudinal research from early childhood until the development of psychosis will help to identify the markers of disease transition and progression (prodrome) and determine when they appear and how stable they are.

## Conclusions

This study reveals that specific psycho-behavioral characteristics were already present during childhood (6–8 years) in adult patients with schizophrenia when retrospectively measured via guardian reports. Although each characteristic is not specific to schizophrenia and overlaps with those already reported in previous research, a combined pattern of withdrawal, problems with thinking, and lack of aggression was extracted from a logistic regression analysis. This combined pattern may be a specific prodromal sign indicative of the start of a transition to psychosis.

## Data Availability

The Child Psychosis-Risk Screening System (CPSS) that incorporates the algorithm obtained by this research is available in an interactive web system at https://nakayama-lab.japanwest.cloudapp.azure.com/prototype/. The data supporting the findings of this study are available from Kyoto women’s university. Restrictions apply to the availability of these data, which were used under license for this study. Data are available from the authors with the permission of Kyoto Women’s University.

## Abbreviations

AUC: Area Under the ROC Curve
CBCL: Child Behavior Checklist
CPSS: Child Psychosis-risk Screening System
DSM-IV-TR: Diagnostic and Statistical Manual of Mental Disorders, Fourth Edition, Text Revision
ROC: Receiver Operating Characteristics
